# Global health policies that support the use of banked donor human milk: a human rights issue

**DOI:** 10.1186/1746-4358-1-26

**Published:** 2006-12-12

**Authors:** Lois DW Arnold

**Affiliations:** 1 National Commission on Donor Milk Banking, American Breastfeeding Institute, 327 Quaker Meeting House Road, East Sandwich, MA 02537, USA

## Abstract

This review examines the role of donor human milk banking in international human rights documents and global health policies. For countries looking to improve child health, promotion, protection and support of donor human milk banks has an important role to play for the most vulnerable of infants and children. This review is based on qualitative triangulation research conducted for a doctoral dissertation. The three methods used in triangulation were 1) writing as a method of inquiry, 2) an integrative research review, and 3) personal experience and knowledge of the topic. Discussion of the international human rights documents and global health policies shows that there is a wealth of documentation to support promotion, protection and support of donor milk banking as an integral part of child health and survival. By utilizing these policy documents, health ministries, professional associations, and donor milk banking associations can find rationales for establishing, increasing or continuing to provide milk banking services in any country, and thereby improve the health of children and future generations of adults.

## Review

Donor milk banking thrives in countries such as Brazil, where there has been a concerted effort at the Health Ministry level to incorporate milk banks into health policy [1]. Its prime mover, Dr. Joao Aprigio Guerra de Almeida, has been honored with the prestigious WHO Sasekawa prize for making an important contribution to his country's overall health by establishing a network of donor human milk banks [2,3]. In countries where donor milk banking is protected, promoted, and supported as an extension of national breastfeeding policies, milk banking is considered a reasonable and effective part of health care delivery for infants and children.

Premature infants who are fed infant formula have a higher risk of developing necrotizing enterocolitis (NEC) than when they are fed human milk, either mother's own milk or banked donor milk [4-6]. In this regard, donor milk banking could be considered preventive "medicine" in the premature population; by reducing the incidence of NEC and optimizing central nervous system development, the premature infant has a better start in life than he would have if fed premature infant formula. The argument has been made [7] that these infants become more productive members of society as adults if their health and neurological potential are maximized through optimal nutrition and appropriate health care from the start. This argument is made despite a general lack of published research on the efficacy of banked human milk because in many parts of the world there is a general belief that human milk in any form is superior to manufactured infant formulas. This is contrary to the pervading philosophy among many health care providers, especially in the US, that infant formula and human milk are equivalent.

If donor milk banking has been incorporated into national public health policy and regulation, (such as France [8,9], Germany [10,11], and the Scandinavian countries [12]) and/or in other countries with socialized medicine, such as Canada and Great Britain, parents do not have to pay out of pocket to receive this service for their infants; it is provided as part of a national health insurance plan to any infant with a medical need. In countries such as the United States, where there is no federal public health policy supporting donor milk banking or regulation of its operations, growth of donor milk banking services has been severely hampered and the recipient population remains underserved.

This review examines the existing international policies from the United Nations, the World Health Organization and UNICEF into which donor milk banking may be specifically integrated. While these policies often do not refer directly to either donor milk banking or breastfeeding, many of them protect, promote and support optimal health. Where the support is indirect, through breastfeeding protection, promotion and support, it can be inferred that donor milk banking "fits" in these policy statements because the support is for a form of human milk delivery. These policies can therefore be interpreted as being supportive of the earliest measures to achieve optimal health, breastfeeding and its adjunct, donor milk banking. Any nation, whether signatory to these agreements or not, thus has a basis for arguing that policies already exist that protect and support donor milk banking and that these policies establish a standard for action. Even if a human rights convention is not ratified or enforced, a precedent has been set and the right remains for that country's citizens.

## Human rights conventions from the United Nations

On December 10, 1948 the United Nations adopted the *Universal Declaration of Human Rights *[13]. Article 25 states that "Everyone has the right to a standard of living adequate for the health and well-being of himself and of his family, including food, clothing, housing and medical care ...". Mothers and children are identified as being entitled to special care and assistance. All children should be provided with the same protection, meaning that sick or premature infants and children must be afforded the same opportunities for achieving good health as a healthy infant or child. While not specified, breastfeeding or the provision of human milk to an infant/child who is unable to nurse is of paramount importance. In the absence of their own mothers' milk, banked donor milk has a role to play in providing for health and well being of this special category of infants and children.

Human rights involve the relationship between a government and its individual citizens. While individuals clearly have some responsibility in terms of their behavior, governments must also take an active role to ensure that the weakest individuals are protected equally as much as the strongest. In developing its human rights conventions, the United Nations places responsibility on governments to protect the rights of its citizens. However, UN conventions do not have the force of law in any country. It is expected that the signatory nations will develop their own legislation to implement the conventions and provide protection of basic human rights in this way [14,15].

Bar-Yam [16,17] has reviewed the United Nations (UN) human rights conventions and placed breastfeeding and human milk in the conventions addressing children's rights, women's rights, and the right to health and health care. The UN conventions on children's rights clearly refer to **all **children and do not distinguish whether children are sick or well. What is inferred is that if a child is sick then the family and government have clear moral obligations to remedy the situation where possible to "provide the highest attainable standard of physical and mental health." [[18], Article 12]. Breastfeeding and human milk, including banked donor milk, take on even greater significance for premature and sick infants and children.

Does "breastfeeding" mean merely consuming human milk or really being fed at the breast? If the baby/mother has the right to feed at the breast, then it is the mother's moral obligation to do so. She needs to take advantage of her right and utilize it. This requires government protection at the national level (e.g., legislation to protect families from formula company marketing tactics or legislation that protects the breastfeeding relationship for working mothers), promotion (e.g., national campaigns to inform the public of the benefits of breastfeeding/hazards of infant formula), and support (e.g., funding of mother-to-mother and peer counselor programs, doulas, and voluntary or professional breastfeeding counselors/consultants that instill self confidence in breastfeeding mothers and provide information, recommendations, and assistance when problems arise). If the mother is unable to feed at the breast, then it is the government that is morally obligated to provide another source of breastfeeding or human milk (e.g., a wet nurse or cooperating mother, where culturally acceptable, or milk from a donor milk bank). If the right is interpreted as the baby's right to be **fed **human milk, then the moral obligation falls on the mother to provide it. In the absence of the mother's breastfeeding or providing her own expressed milk, it falls to the government to provide human milk in some other way, such as through a milk bank [16].

In 1967, the UN adopted the *International Covenant on Economic, Social and Cultural Rights *[18]. Article 12 states that all individuals have the right to the "highest attainable standard of physical and mental health." Countries need to take steps to lower infant mortality and ensure the healthy development of the child [[17], p. 32]. Breastfeeding and human milk fit into this convention well. There is a definitive relationship of infant formula feeding with an increase in infant mortality rates and poorer infant and child health outcomes [19,20]. Banked donor milk has been used to reduce morbidity and infant mortality. Donor milk feedings reduce the number of days of hospitalization required by the presence of NEC. (According to Bisquera et al, [21] a resolved case of NEC extends the hospital stay by approximately two weeks.) Additionally, if fewer cases of NEC result, then fewer surgeries are required to remove necrotic portions of the gut and fewer individuals therefore have surgically-induced short bowel syndrome and life-long malabsorption problems [22].

*The Convention on the Elimination of All Forms of Discrimination Against Women *was adopted in 1981. This convention recognizes that certain groups require special protection. According to Article 5b, "...the interest of the children is the primordial consideration in all cases." [23]. Family education becomes an important factor so that adult family members understand the importance of motherhood and that mothers raise future members of the society and culture. Pregnant women and mothers should, therefore, be afforded special protection so that they might care for their children in an optimal way. If the interests of the child have top priority, providing them optimal nutrition when they most need it should also be a priority. Breastfeeding and banked donor milk fit here as needing special protection.

Several articles in the *Declaration and Convention on the Rights of the Child *[24] also apply to donor milk banking. Article 3 reiterates that the best interests of the child are primary. This belief has previously been expressed in the earlier declarations relating to children's rights as well as other UN conventions [12,18,25]. Article 18 specifies that governments should provide assistance to families through institutional and legislative support. [[17], pp. 37–38]. In relation to breastfeeding and the use of human milk this means that a country has a responsibility for protecting breastfeeding through legislation, including legislation to restrict marketing practices of infant formula companies. If other forms of infant nutrition are needed, the manufacture of these foods should be regulated for safety and adequacy. In terms of donor milk banking, this means that governments need to ensure that human milk alternatives to infant formula are provided and that there exists quality control and governmental or other legislative oversight to ensure that human milk obtained from other mothers can be fed safely to an unrelated infant/child.

Breastfeeding is addressed directly in the Convention on the Rights of the Child in Article 24. The article begins by saying that "States Parties recognize the right of the child to the enjoyment of the highest attainable standard of health and to the facilities for the treatment of illness and rehabilitation of health." [24]. Because donor milk is primarily used as therapeutic nutrition for infants whose health requires improvement, donor milk banks become facilities that are an integral part of the process of treatment and rehabilitation. Governments therefore need to actively become involved in the creation of these facilities and/or their operation. Various ways that governments can do this are to: make national policy statements about the importance of donor milk banking; provide seed money or continuous funding for the establishment and operation of donor milk banks; provide regulatory and research support as well as expert consultation on standards of operation; and implement the *International Code of Marketing of Breast-milk Substitutes *[26] so that donor milk can compete fairly with commercially available manufactured breast milk substitutes.

Article 24 continues by stating that breastfeeding is an activity for the whole society (Section 2e). Mothers are not mandated to breastfeed, but governments are mandated to educate all mothers and parents so that they can make informed choices. [[17], pp 39–40] By extension, this means that parents should also be educated about the uses of banked donor milk and its benefits, so that they know about this option and can request it if necessary.

## UNICEF policy statements encouraging the use of human milk for fragile infants

UNICEF has set forth four principles for improved child survival in its "*GOBI Initiative*." First articulated by James Grant in his 1982 State of the World's Children address, these principles have become the foundation for primary health promotion and illness prevention programs throughout the world [27]. The four principles are:

• **G**rowth

• **O**ral rehydration

• **B**reastfeeding

• **I**mmunization

Despite the "arid" implications of the acronym, GOBI has been a fertile basis for improved infant and child health throughout the world.

Breastfeeding is the foundation of the GOBI campaign (even though it is third on the list) because it is an integral part of the other components. Breastfeeding provides most infants with adequate growth for at least the first six months of life when practiced exclusively and is critical for continued optimal growth as complementary foods are added to the diet [28]. Because human milk is approximately 87 percent water [29], it provides adequate hydration in even the warmest climates and can prevent dehydration during bouts of diarrhea [30]. The immune properties of colostrum and human milk provide early immunization and protection against disease to the nursing infant. Banked donor milk provides similar immune protection to premature or ill infants, species-specific nutrition to foster adequate growth, and has been used medicinally to treat cases of chronic diarrhea and keep the baby hydrated [31,32].

**Figure 1:**
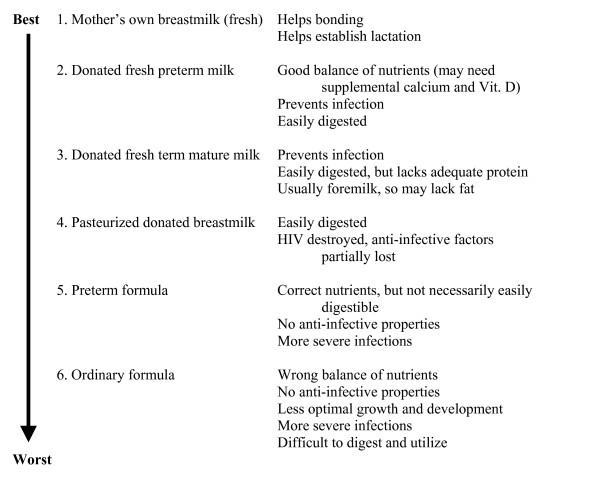
Milk for Low Birth Weight Babies: WHO Hierarchy of Feeding Choices [14].

Breastfeeding at the breast is number one in the World Health Organization's hierarchy of infant feeding choices for infants who have the ability to breastfeed. For premature and sick infants it may be that actual breastfeeding is impossible for a variety of reasons (e.g., gestational age, oromotor abnormalities) in which case the mother's fresh expressed milk becomes the choice. Figure 1 is a diagram of the *Hierarchy of Feeding Choices for Low Birth Weight Infants *[Savage, personal communication, 1998; [14]]. Publication of this hierarchy has become mired in international HIV politics, particularly in relation to the choice of fresh milk donated by a biologically unrelated mother (options 2 and 3). Countries such as the United States have difficulties with these two options because of the risk of disease transmission, in particular HIV. In the US the risk of disease transmission outweighs the benefits of fresh human milk from an unrelated donor. Even though pasteurization negatively affects some of the protective factors in donor milk and may increase infection rates slightly, the use of infant formula with NO protective effect dramatically increases infection rates [33]. In developing countries, where human milk substitutes carry a much higher risk of infant mortality than in a developed country, and where pasteurization of the donated milk is not widely available, the two "raw milk" alternatives in the feeding hierarchy might be preferable because the risk of potential disease transmission and death from the raw donor milk is outweighed by the much higher risk of death from the use of infant formula. Newer guidelines relating to wet nurses in these countries recommend that all wet nurses be screened for HIV [34]. The use of pasteurized donor milk in populations of infants orphaned by HIV has proved efficacious in one setting in South Africa [35]. In the absence of the mother's own milk and in light of the high risk of infant formula feeding in countries with a high rate of HIV/AIDS, donor milk collected and processed in a systematic way through donor screening, bacteriological testing of the milk, and pasteurization may protect a large segment of the population and foster child growth and development that will assist individuals in becoming future contributing members of society.

*The Baby-Friendly Hospital Initiative *(BFHI), a joint UNICEF and WHO initiative established in 1989 [36], also contains numerous opportunities within its foundation, the *Ten Steps to Successful Breastfeeding *(see Table 1), for the implementation of donor milk banking practices and the use of banked donor milk. Policies and procedures for ensuring that a mother is taught how to establish and/or maintain her milk supply during separation from her baby should be in place (Step 1). Policies relating to the use of donor milk in the hospital, how to store, freeze and thaw it as appropriate, and how to document its use, etc. would fall under this step as well. All staff that come in contact with these mothers and their babies must be trained so that they support the breastfeeding process and the use of human (donor) milk (Step 2). It would naturally follow that if staff members are being trained on all policies and there is a policy on donor milk, then training on that policy would also be conducted. Step 3 (relating to prenatal education) should include education for parents about the availability of donor milk and its benefits in the event that the mother's own milk is unavailable. This education is a natural extension of educating the parents about the benefits of breastfeeding. Initiation of breastfeeding within the first hour of life (Step 4) could be interpreted that first feedings should be of banked donor milk if mother's own milk is not available and should be given early as minimal enteral feeds for premature infants or as oral care. Step 5 could be interpreted as involving milk banks as well. In this step, mothers must be aided in the establishment and maintenance of lactation through milk expression even when they are separated from their infant. Mothers should be informed of the opportunity for becoming milk donors if they establish an ample supply and have an excessive amount of milk, or, conversely, be informed of the availability of donor milk as back-up for their own supply if the latter should falter. Mothers should always be informed of the importance of their own milk in feeding their own infant as well as its potential for feeding other infants when donated to a milk bank.

**Table 1:** The Ten Steps to Successful Breastfeeding [36]

Every facility providing maternity services and care for newborn infants should:
1. Have a written breastfeeding policy that is routinely communicated to all health care staff.
2. Train all health care staff in skills necessary to implement the policy.
3. Inform all pregnant women about the benefits and management of breastfeeding.
4. Help all mothers initiate breastfeeding within the first hour of birth.
5. Show mothers how to breastfeed and maintain lactation even if separated from their infants.
6. Give newborn infants no food or drink unless medically indicated.
7. Practice rooming in: allow mothers and infants to stay together 24 hours a day.
8. Encourage breastfeeding on demand.
9. Give no artificial teats or pacifiers.
10. Foster the establishment of breastfeeding support groups and refer mothers to them on discharge.

Additionally, many neonatal intensive care units (NICUs) and special care nurseries (SCNs) rely on free supplies of "specialty" infant formulas for premature or ill infants that may be considerably more expensive than infant formulas used for feeding healthy term infants. Within the *International Code of Marketing of Breastmilk Substitutes *and subsequent resolutions, all infant formulas should be paid for and, within the criteria for Baby Friendly status, used only when medically indicated. Banked donor milk certainly has a role to play here when the mother's own expressed milk is not available (Step 6) and should be used in preference to infant formula whenever possible, as per the hierarchy of feeding choices.

## WHO/UNICEF policies specific to donor milk banking

Over the years WHO has had a remarkably consistent policy with regard to human milk banking. In 1979, WHO and UNICEF issued a joint resolution on infant and young child feeding that was fully endorsed by the World Health Assembly in 1980. The "first alternative" when a mother is unable to breastfeed should be human breast milk, using banked donor milk where appropriate and available [37]. In 1992 banked donor milk was included as an acceptable feeding alternative when the biological mother tests positive for the human immunodeficiency virus (HIV) [38]. In 1998, and reaffirmed in 2003, banked donor milk was presented as an option in a publication on HIV and infant feeding [39]. As mentioned previously, WHO has also affirmed the importance of donor milk banking in the awarding of the prestigious Sasakawa Prize for 2001 to Dr. Joao Aprigio Guerra de Almeida of Brazil for his work in organizing the largest and most important donor milk banking system in the world.

Most recently, in 2002 the World Health Assembly unanimously endorsed the *Global Strategy for Infant and Young Child Feeding*, which recommends banked donor milk as an option when the infant cannot breastfeed and/or the mother's own expressed milk is unavailable. Section 18 of the WHO/UNICEF Global Strategy on Infant and Young Child Feeding [40] states:

The vast majority of mothers can and should breastfeed, just as the vast majority of infants can and should be breastfed. Only under exceptional circumstances can a mother's milk be considered unsuitable for her infant. For those few health situations where infants cannot, or should not, be breastfed, the choice of the best alternative – expressed breast milk from an infant's own mother, breast milk from a healthy wet nurse or a human-milk bank, or a breast milk substitute fed with a cup, which is a safer method than a feeding bottle and teat – depends on individual circumstances. [[40], p. 10]

The "emerging policy framework" of this statement builds on the existing policy documents of the *International Code of Marketing of Breastmilk Substitutes *[26] the Innocenti Declaration on the Protection, Promotion and Support of Breastfeeding [41] and the Baby-Friendly Hospital Initiative [36]. But it goes further in emphasizing that countries need to develop and implement *comprehensive *national policies on infant and young child feeding.

## Conclusions: whose responsibility is it?

What makes the Global Strategy on Infant and Young Child Feeding [40] so important is the steps taken to delineate responsibilities and obligations that various parties have. Governments, health professional associations, non-governmental organizations (NGOs) including community-based support groups, commercial enterprises, employers, and other groups all have responsibilities for working as partners and making the Global Strategy successful. Governments are charged with formulating, implementing, monitoring, and evaluating a comprehensive national policy related to infant and young child feeding. National coordination and political commitment "at the highest level" are required to make the Global Strategy a success. Regional and local governments should contribute to this process. Governments also need a plan of action and should develop goals and objectives, develop a timeline for its implementation and allocate responsibilities for parts of the plan. Then governments must identify and commit adequate resources to the endeavor, part of which is to ensure that donor milk banking services continue to exist as a backup for when a mother's own milk is unavailable.

It is equally important for professional organizations to advocate for donor milk banks and to use their influence to apply pressure on governments. Health professional bodies, including medical schools, schools of public health, and institutions that train other allied health care workers dealing with mothers and children need to make sure that the training being given adequately covers breastfeeding and lactation management, including health care providers' responsibilities under the *International Code of Marketing of Breast-milk Substitutes *[26]. Health care providers should also be prepared to implement and maintain the Baby-Friendly Hospital Initiative and the Ten Steps to Successful Breastfeeding in hospitals [36], using donor milk when there is a medical indication for supplementation and the mother's own milk is unavailable.

NGOs have the responsibility for developing community-based interventions that support breastfeeding and adequate food and nutrition and adequate paid maternity leave. They also have the responsibility for interfacing with and creating linkages with the health care system. Beyond providing safe foods that meet standards of the *Codex Alimentarius *and the *Codex Code of Hygienic Practice for Foods for Infants and Children*, commercial enterprises should obey the tenets of the *International Code of Marketing of Breast-milk Substitutes *and subsequent resolutions and monitor their marketing practices to comply with the Code. Employers should work with trade unions to ensure that women can continue to breastfeed or express milk safely in the workplace in order to extend the duration of time when infants receive human milk. Other groups such as child care organizations and facilities, the mass media, and educational systems have a role to play in supporting breastfeeding and depicting breastfeeding as the norm for infant and young child feeding. **All **groups, by extension, have the responsibility to protect and support donor milk banking as an integral component of protecting, promoting and supporting breastfeeding in order to ensure optimal health for all infants and children, regardless of their health status at birth.

As of 1999–2000, Brazil had over 150 donor milk banks that collected and processed more than 218,000 liters of donated human milk that was fed to over 300,000 premature and low birth weight babies. The provision of this milk is estimated to have saved the Brazilian Ministry of Health approximately US$540 million that year. Trained firefighters go to donor mothers' homes to pick up the collected milk, underscoring the importance of breastfeeding to the entire country. More recent statistics indicate that there are now over 186 milk banks in Brazil [42]. The number of liters dispensed, the number of infants who have benefited from the provision of donor milk, and the savings to the ministry of health can only have increased, making Brazil's donor milk banking system one that other countries should emulate. What is so unique about Brazil is that in promoting, protecting and supporting donor milk banking they have also found ways to promote, protect and support breastfeeding and make breast milk feeding the cultural norm. Protecting breastfeeding then promotes and protects donor milk banking.

Other countries should learn a lesson from Brazil. By supporting the importance of donor milk banks and the clinical use of donor milk, the healthy future of an entire country could be improved. It is the right of **all **children to be included in this healthy future through ensuring that banked human donor milk is accessible to all who need it. Everyone has an ethical obligation to promote, protect and support donor milk banking, from governments, to professional associations, to individuals.

## Competing interests

The author(s) declare that they have no competing interests.

## Authors' contributions

This work is solely the work of the single author.
